# Impact of SAfinamide on Depressive Symptoms in Parkinson’s Disease Patients (SADness-PD Study): A Multicenter Retrospective Study

**DOI:** 10.3390/brainsci11020232

**Published:** 2021-02-13

**Authors:** Esteban Peña, Carmen Borrué, Marina Mata, Juan Carlos Martínez-Castrillo, Araceli Alonso-Canovas, Juan Luis Chico, Lydia López-Manzanares, Marcos Llanero, Jaime Herreros-Rodríguez, Alberto Esquivel, Teresa Maycas-Cepeda, Cristina Ruíz-Huete

**Affiliations:** 1 Neurology Department, Hospital La Moraleja, 28050 Madrid, Spain; 2 Neurology Department, Hospital Infanta Sofía, 28703 Madrid, Spain; carmenborrue@hotmail.com (C.B.); mmataal@yahoo.es (M.M.); 3 Neurology Department, Hospital Ramón y Cajal, 28034 Madrid, Spain; jcmcastrillo@gmail.com (J.C.M.-C.); aracelialcan@yahoo.es (A.A.-C.); juanluis.chico.garcia@gmail.com (J.L.C.); 4 Neurology Department, Hospital La Princesa, 28006 Madrid, Spain; lydialopez@hotmail.com; 5 Neurology Department, Complejo Hospitalario Ruber Juan Bravo, 28006 Madrid, Spain; mllanero@gmail.com; 6 Neurology Department, Hospital Infanta Leonor, 28031 Madrid, Spain; hrinvest@hotmail.com (J.H.-R.); alberto.esquivel@salud.madrid.org (A.E.); 7 Neurology Department, Hospital Quironsalud Madrid, 28223 Madrid, Spain; tmaycas@gmail.com; 8 Neurology Department, Clínica del Rosario, 28006 Madrid, Spain; ruizhuete@yahoo.es

**Keywords:** Parkinson’s disease, depression, serotonin syndrome, antidepressants, safinamide, motor symptoms

## Abstract

Background: We aimed to assess the effects of safinamide on depression, motor symptoms, and the serotonin syndrome related to its co-administration with antidepressants in patients with Parkinson’s disease (PD). Methods: We retrospectively analyzed the data of patients at 1 and 3 months of follow-up compared to baseline. Results: *n* = 82 (safinamide 50 mg = 22, 100 mg = 60, with antidepressants = 44). First, we found improvement in depression (Hamilton Depression Rating Scale: −6 ± 5.10 at 1 month and −7.27 ± 5.10 at 3 months, *p* < 0.0001; Patient Global Impression of Improvement Scale: 60.3% and 69.5% of patients at 1 and 3 months reported some improvement). Second, safinamide improved the daily life activities and motor symptoms/motor complications (Unified Parkinson’s Disease Rating Scale (UPDRS-II): −2.51 ± 6.30 and −2.47 ± 6.11 at 1 and 3 months, *p* < 0.0001; III: −3.58 ± 8.68 and −4.03 ± 8.95 at 1 and 3 months, *p* < 0.0001; IV: −0.61 ± 2.61 and −0.8 ± 2.53 at 1 and 3 months, *p* < 0.0001). Third, 7.31% and 8.53% of patients developed non-severe adverse events related to safinamide at 1 and 3 months. Serotonin syndrome was not observed in the patients treated with antidepressants; some isolated serotonin syndrome symptoms were reported. Conclusions: Safinamide could be useful for treating depression in PD; it was effective for motor symptoms and motor complications and safe even when co-administered with antidepressants.

## 1. Introduction

Safinamide is a reversible and selective monoamine oxidase B inhibitor (MAOIB) and glutamate release modulator [1]. Several trials have demonstrated that in advanced Parkinson’s disease (PD), safinamide significantly improves “ON” time without causing troublesome dyskinesia, reduces “OFF” time, and improves scores on the Unified Parkinson’s Disease Rating Scale (UPDRS), improving quality of life [2,3,4,5,6]. Thus, it is approved for the treatment of mid-to-late fluctuating PD patients as an add-on therapy alongside stable doses of levodopa alone or in combination with others drugs. However, few studies have evaluated the role of safinamide in real clinical practice [7].

Depression is not only one of the most common non-motor symptoms in PD, with a prevalence around 30–35%, but it is also the main determinant of quality of life [8,9]. Although the pathophysiology of depression in PD is complex, dopamine and glutamate disorders could be involved [8,10]. Accordingly, it has been demonstrated that dopaminergic therapy, including MAOIBs, can improve depressive symptoms in PD patients [11,12,13,14]. In fact, some authors recommend that in Parkinson’s disease patients with depression, it could be useful to modify dopaminergic therapy before to add antidepressants [8]. Furthermore, drugs that inhibit abnormal presynaptic glutamate release such as lamotrigine or riluzole are considered mood stabilizers [15]. Thus, considering the dual mechanism of action of safinamide as a glutamatergic modulator and dopaminergic stimulator, we hypothesized that safinamide could be useful for improving depression in PD.

Concerns exist regarding the safety of combining MAOBIs with antidepressants, because of the risk of the potentially fatal serotonin syndrome, although serotonin syndrome is rarely induced by MAOBIs such as selegiline and rasagiline [16,17,18,19,20]. However, there are no studies assessing serotonin syndrome in patients concomitantly treated with safinamide and antidepressants.

The aim of this study was to assess the effect of safinamide on depression in PD patients. The secondary goals were to assess the tolerability of safinamide in real clinical practice, with a special focus on serotonin syndrome in PD patients concomitantly treated with safinamide and antidepressants, and to assess the effect of safinamide on motor symptoms, motor complications, and daily life activities for PD patients in real clinical practice.

## 2. Materials and Methods

### 2.1. Study Design and Population

This was a multicenter, observational, retrospective study based on real clinical practice. Up to March 2020, researchers from the movement disorder units of 13 different hospitals selected PD patients from medical history databases fulfilling the following inclusion criteria: aged over 18 years, with a PD diagnosis (according to MDS clinical diagnostic criteria [21]) and depression diagnosis (a Hamilton Depression Rating Scale based on 17 items, HAMD-17, >14 [22]), and being treated with safinamide within labeled use (according to the terms of the marketing authorization), with full clinical assessments at baseline, one month (when available) and three months after the onset of safinamide treatment. The clinical data required were demographic data, HAMD-17 scores, Patient Global Impression of Improvement Scale (PGI-I) scores with respect to depressive symptoms, UPDRS scores, concomitant treatment with antidepressants and other anti-Parkinsonian drugs, and registered adverse events, with a special focus on serotonin syndrome symptoms. The main exclusion criteria were PD-associated dementia and patients who underwent other major changes in antidepressant or anti-Parkinsonian drug treatments during the follow-up period.

The sample was divided according to safinamide dose into 50 and 100 mg/day groups and also according to antidepressant use (safinamide-only vs. safinamide-plus-antidepressants group) to assess potential serotonergic adverse events.

The primary outcome measure for the antidepressant effect was the HAMD-17 scores at 1 and 3 months. The PGI-I scores related to depressive symptoms were considered as the secondary outcome measure.

As for daily life activities, motor symptoms, and motor complications, changes in UPDRS Parts II, III, and IV at 1 and 3 months (from baseline) were compared. PD patients were assessed in ON-medication states.

To test for serotonin syndrome, we followed previously reported methods [18]. Patients of both the safinamide-only and safinamide-plus-antidepressants groups were compared for 15 symptoms linked to serotonin toxicity: (a) major symptoms: confusion, emotional lability, fever, sweating, and myoclonus; (b) minor symptoms: agitation, sleep disorders, nervousness, tachycardia, hyperventilation, dyspnea, diarrhea, hypertension, hypotension, and ataxia. These symptoms were registered whenever present, regardless of whether the investigator considered them to be drug related or not. Serotonin syndrome was diagnosed in patients who had combinations of at least 3 major symptoms. We chose this definition because it was considered more inclusive than those definitions where minor symptoms were included [18].

Levodopa equivalent daily dose (LEDD) was calculated according to previous reports [23,24].

### 2.2. Statistical Analyzsis

The demographic and clinical data are shown as means (standard deviations), ranges, or relative frequencies. The PGI-I scores are shown as relative frequencies. Comparisons between baseline and 1 and 3 months for the variables HAMD-17 and UPDRS were conducted using the Student’s t-test for paired data. The frequencies of serotonin syndrome symptoms were compared between the safinamide-only and safinamide-plus-antidepressants groups with the Fisher’s exact test. *p* values < 0.05 were considered statistically significant.

## 3. Results

We enrolled 82 patients with a minimum follow-up period of 3 months; 78 of them had available data at 1 and 3 months. Twenty-two patients (26.8%) were treated with 50 mg of safinamide, and sixty (73.2%) were treated with 100 mg. Of the 82 patients recruited, 44 (53.7%) received concomitant treatment with antidepressants. The demographic and clinical data at baseline are shown in Table 1.

The doses of anti-Parkinsonian drugs remained largely stable throughout the study: LEDDs were 810.2 (368.45) mg at baseline, +26,07 (424.10) mg at 1 month, *p* = 0.3763 (Student’s t-test for paired data), and −4.13 (376,11) mg at 3 months (*p* = 0.3763, Student’s t-test for paired data). Furthermore, in the group of patients concomitantly treated with safinamide and antidepressants, the doses of antidepressant drugs did not change during the follow-up period. The antidepressants prescribed and their doses are listed in Table 2.

### 3.1. Effect of Safinamide on Depression in PD Patients

**Figure 1:**
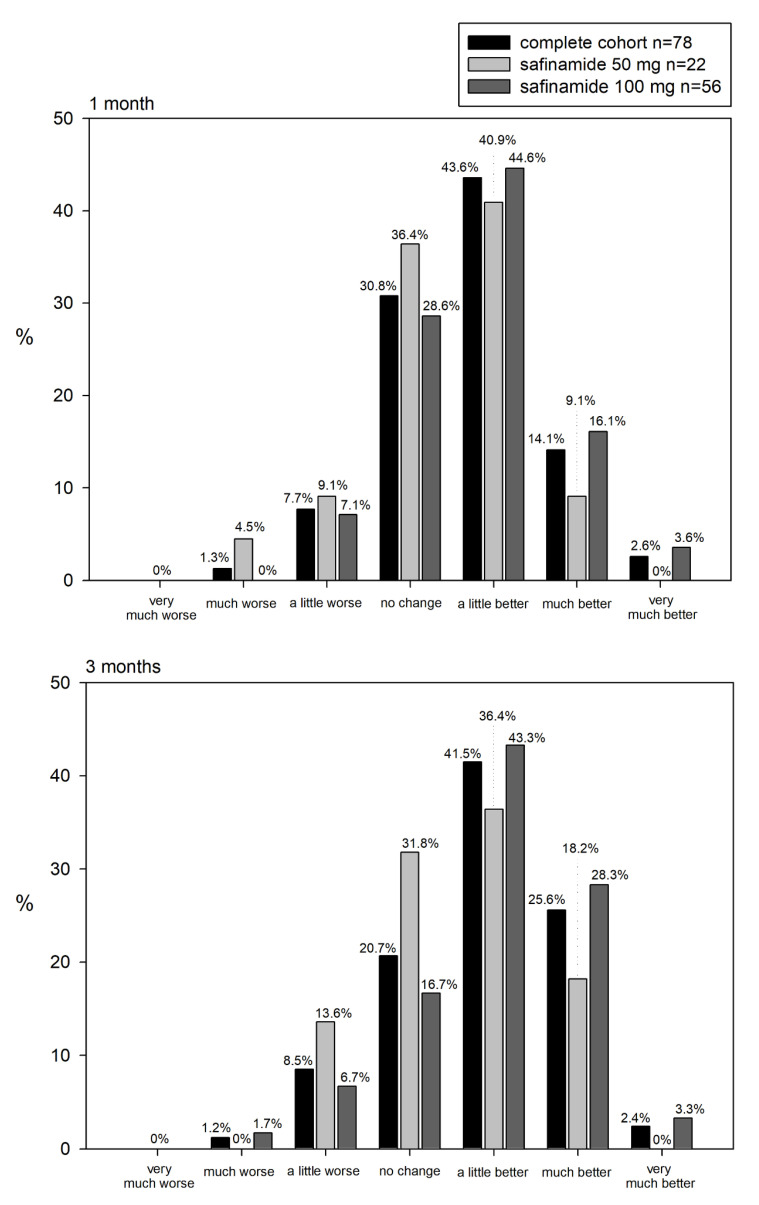
Patient Global Impression of Improvement Scale scores at 1 and 3 months.

The primary outcome measure for the antidepressant effect (the HAMD-17 score) showed significant improvements of −6 (5.10) points at 1 month and −7.27 (5.10) points at 3 months (*p* < 0.0001). Furthermore, there was a significant fall in the HAMD-17 scores at 1 and 3 months for both doses, although a tendency toward greater reductions with 100 vs. 50 mg was observed (Table 3). In the same line, 60.3% of patients at 1 month and 69.5% at 3 months reported some improvement in their depressive symptoms according to the PGI-I scale (Figure 1). Overall, the perception of improvement according to the PGI-I scale was higher with 100 than 50 mg of safinamide (see Figure 1).

### 3.2. Safinamide on Motor Symptoms, Motor Complications, and Daily Life Activities in Real Clinical Practice

In the analysis of the complete cohort, we observed a significant improvement in UPDRS Part II (−2.51 (6.30) and −2.47 (6.11) points at 1 and 3 months respectively, *p* < 0.0001, Table 3) and UPDRS part III (−3.58 (8,68) and −4.03 (8,95) points at 1 and 3 months, respectively, *p* < 0.0001, see Table 3). UPDRS Part IV also showed mild but significant improvements of −0.61 (2.61) and −0.8 (2.53) points at 1 and 3 months, *p* < 0.0001 (Table 3). However, only 100 mg of safinamide significantly improved UPDRS Parts II, III, and IV (see Table 3).

### 3.3. Serotonin Syndrome in Patients Concomitantly Treated with Antidepressant Drugs: Other Adverse Events

The relative frequencies of the symptoms related to serotonin syndrome in the patients concomitantly treated with safinamide and antidepressants vs. the patients only treated with safinamide are shown in Table 4. Overall, these symptoms were present in a low proportion of patients in both groups. Only “sleep disorders” (16.7% vs. 5.1% at 1 month, *p* = 0.053, and 15.9% vs. 4.9% at 3 months, *p* = 0.054) and “nervousness” (19.2% vs. 5.1% at 1 month, *p* < 0.05, and 15.9% vs. 6.1% at 3 month, *p* = 0.108), both minor symptoms, were notably more frequent in the safinamide + antidepressant group, although significant differences were only found in “nervousness” at 1 month (Table 4). According to the established criteria, there were no patients with serotonin syndrome in our cohort. However, in two patients, serotonin toxicity symptoms, although not severe, led to discontinuation of the drug (in one case, safinamide; in another, duloxetine). The first patient was a 68-year-old man treated with safinamide at 50 mg/day plus sertraline at 50 mg/day who developed confusion, sleep disorders, and diarrhea, and the symptoms improved upon the withdrawal of safinamide. The second patient was a 90-year-old woman with a complex condition of advanced PD and chronic pain. She was treated with safinamide at 50 mg/day and duloxetine at 30 mg/day, developing confusion, myoclonus, sleep disorders, and nervousness. These symptoms improved with the withdrawal of duloxetine. Importantly, this patient was concomitantly treated with tramadol at 37.5 mg/day, since some opioids such as tramadol can inhibit the reuptake of serotonin by inhibiting the serotonin transporter, which increases the serotonergic effect.

Finally, 7.31% of the patients developed other safinamide-related adverse events not associated with serotonin syndrome at 1 month, and 8.53% did so at 3 months. These were nausea (two patients, 2.43%), dyskinesia (one patient, 1.21%), fatigue (one patient, 1.21%), dizziness (one patient, 1.21%), and blurred vision (one patient, 1.21%). None were judged as severe.

## 4. Discussion

Safinamide, with a dual effect as a glutamatergic modulator and dopaminergic stimulator, could theoretically be useful in the treatment of depression in PD patients. However, heterogeneous results have been reported from clinical trials. In a study with early PD patients (study 015), safinamide (in 100 or 200 mg doses) did not improve Hamilton scale scores compared with placebo [25]. Additionally, in studies on mid-to-late PD patients such as 016 and SETTLE, neither 50 nor 100 mg of safinamide resulted in significant changes in Hamilton score vs. placebo [2,4]. However, these results were not conclusive, considering that patients with depression were excluded from studies 015, 016, and SETTLE, meaning that the baseline Hamilton scale scores were low in those studies [2,4,25]. By contrast, statistically significant differences in GRID Hamilton Rating Scale for Depression (GRID-HAM-D) scores were realized with 100 mg doses of safinamide in an 18-month extension of study 16 (study 018) [3]. In addition, the pooled analysis of studies 016 and 018 showed significant long-term improvements in the safinamide (100 mg/day) group vs. placebo, in terms of both the GRID-HAM-D and the “Emotional well-being” domain of the PDQ-39 as well as the proportions of patients reporting depression as an adverse event [26]. In the same line, an observational study showed that 100 mg/day of safinamide significantly improved scores on the non-motor symptoms scale for PD domains related to mood [27]. In agreement with these findings, our real clinical experience showed objective and subjective improvements in depression according to the HAMD-17 and PGI-I scales in PD patients. Note that by definition, the baseline HAMD-17 scores in our cohort were greater than 14, in contrast to the much lower baseline Hamilton scale scores of the studies 015, 016, and SETTLE [2,4,25]. Therefore, we suggest that safinamide could be useful in the treatment of depression in PD.

Although robust improvements in depression in our cohort were observed with both doses, 100 mg seems to be more effective. MAOB has been shown to be almost completely inhibited by 50 mg/day of safinamide [28], so the extra benefit observed with 100 mg/day may be mostly due to nondopaminergic mechanisms. Therefore, the enhanced benefit for depressive symptoms observed in our study with 100 mg of safinamide not only supports a nondopaminergic role in the improvement of depression in PD patients but also implies an interesting difference between safinamide and other dopaminergic drugs that lack these nondopaminergic effects. Nevertheless, the potential biases and insufficient sample size in the 50 mg safinamide group, as discussed below, preclude definite conclusions in this regard.

Based on the UPDRS analysis, our real clinical practice study confirms that safinamide may improve motor symptoms, motor complications, and daily life activities in PD patients, which is in agreement with previous reports [2,3,4,5,29,30]. Supporting these findings, a recent meta-analysis that evaluated both motor function and the activities of daily life in PD patients treated with safinamide suggested that the drug not only improves scores for UPDRS Parts II and III over placebo [31] but also improves motor function, motor fluctuations, and quality of life in PD [31]. However, we found important differences between the 100 and 50 mg doses of safinamide: 100 mg led to significant improvements in UPDRS Parts II, III, and IV, while 50 mg did not result in any significant differences. In previous studies, safinamide at 50 mg/day also did not lead to significant differences in UPDRS II and IV [2,3,5], although an improvement in UPDRS Part III was observed [2,3,5,7], which is in contrast with our results. This difference may be related to the low number of patients in our 50 mg safinamide group (*n* = 22, 26.8%) and, possibly, a selection bias for patients kept on a low dose of safinamide in the medium term; for most patients, it is only a titration dose used for a short period. Regardless, other studies have more often observed benefits from safinamide at 100 mg than 50 mg/day doses [2,6].

We found safinamide to be well tolerated in real conditions, even when co-administered with antidepressants, which is in consonance with previous reports [30,32]. Overall, the relative frequencies of major and minor symptoms associated with serotonin syndrome were low, without significant differences between the safinamide-only and safinamide + antidepressants groups. Only, “sleep disorders” and “nervousness” were notably more frequent in the safinamide + antidepressant group, but significant differences were only observed in “nervousness” at 1 month. Furthermore, these were minor symptoms, not serious, and potentially linked to the depression and antidepressants themselves. These findings are similar to previous reports on rasagiline [18]. Finally, according to the established criteria, no patient in our cohort developed serotonin syndrome, which is similar to in previous studies with rasagiline and safinamide [17,30]. However, two patients withdrew from the treatment due to major symptoms, although they were not severe. Even though safinamide is safe in patients older than 75 years [30], an advanced age and concomitant treatment with opioids are likely to have played a role in these cases. It is important to explain here that some opioids such as tramadol can inhibit the reuptake of serotonin by inhibiting the serotonin transporter, and therefore, they should also be considered serotonergic drugs [33]. Thus, our experience suggests that the co-administration of safinamide and antidepressants is safe, although caution is warranted, especially for the elderly, for whom we recommend avoiding other serotonergic drugs, for instance, opioids as tramadol, using doses as low as possible, and closely monitoring for adverse events [20].

We must acknowledge several limitations of our study. First, it was an observational retrospective study where comparisons were made with respect to baseline, so it lacked a control cohort without safinamide treatment, and there was a possible selection bias related to non-controlled withdrawals, which could have led to the overestimation of the results with respect to the population. This bias is frequent in retrospective designs. Second, the observation period established in the design was short, and the final sample size was small; both of these were due to difficulties in obtaining the required data in a retrospective manner. Third, for reasons explained above, we could not draw definitive conclusions regarding the differential effects of safinamide at 50 mg on motor and non-motor symptoms. Future prospective studies or clinical trials with control groups could overcome these limitations.

## 5. Conclusions

Safinamide could be useful for the treatment of depression in PD. In real clinical conditions, safinamide seems to be efficacious in improving motor symptoms, motor complications and daily life activities. Greater benefits for both depression and motor symptoms appear to be realized with 100 mg/day doses. Safinamide seems to be well tolerated in real clinical practice, even when co-administered with antidepressant drugs, but it should still be used with caution.

## Figures and Tables

**Table 1:** Demographic and clinical data at baseline (*n* = 82).

n	n (%)	Complete cohort	82
		Safinamide-only group	38 (46.3%)
		Safinamide + antidepressants group	44 (53.7%)
Age (years)	Mean (SD) [range]	Complete cohort	68.33 (11.41) [41–90]
		Ssafinamide-only group	70.13 (9.83) [41–87]
		Safinamide + antidepressants group	66.77 (12.51) [42–90]
Gender (male/female)	n (%)/n (%)	Complete cohort	37 (45.1%)/45 (54.9%)
		Safinamide-only group	21 (55.3%)/17 (44.7%)
		Safinamide + antidepressants group	16 (36.4%)/28 (63.6%)
Disease duration (years)	Mean (SD)	Complete cohort	8.67 (8.55)
		Safinamide-only group	7.84 (9.65)
		Safinamide + antidepressants group	9.39 (7.51)
UPDRS			
I	Mean (SD)	Complete cohort	4.56 (1.82)
		Safinamide-only group	3.82 (1.90)
		Safinamide + antidepressants group	5.20 (1.49)
II	Mean (SD)	Complete cohort	13.59 (6.67)
		Safinamide-only group	13.55 (7.07)
		Safinamide + antidepressants group	13.61 (6.38)
III	Mean (SD)	Complete cohort	22.91 (8.68)
		Safinamide-only group	22.47 (9.90)
		Safinamide + antidepressants group	23.30 (7.57)
IV	Mean (SD)	Complete cohort	3.51 (2.83)
		Safinamide-only group	2.61 (2.52)
		Safinamide + antidepressants group	4.30 (2.87)
HAMD-17	Mean (SD)	Complete cohort	19.49 (4.03)
		Safinamide-only group	18.39 (3.58)
		Safinamide + antidepressants group	20.43 (4.20)
LEDD (mg)	Mean (SD)	Complete cohort	810.26 (368.45)
		Safinamide-only group	681.25 (218.15)
		Safinamide + antidepressants group	921.68 (432.86)

UPDRS: Unified Parkinson’s Disease Rating Scale. HAMD-17: Hamilton Depression Rating Scale based on 17 items. LEDD: Levodopa equivalent daily dose. SD: Standard deviation.

**Table 2:** List of antidepressants concomitantly prescribed with safinamide.

Antidepressant Drug	Range of Doses (mg/day)	*n* (%)
DULOXETINE	30–120	11 (25.0%)
ESCITALOPRAM	5–15	7 (15.9%)
MIRTAZAPINE	15–30	5 (11.4%)
SERTRALINE	50–100	5 (11.4%)
VENLAFAXINE	75–150	5 (11.4%)
TRAZODONE	50–100	4 (9.1%)
AMITRIPTILINE	25	1 (2.3%)
CLORIMIPRAMINE	25	1 (2.3%)
BUPROPION	150	1 (2.3%)
CITALOPRAM	20	1 (2.3%)
PAROXETINE + AMITRIPTILINE	10 + 25	1 (2.3%)
VENLAFAXINE + MIRTAZAPINE	75 + 15	1 (2.3%)
VORTIOXETINE	10	1 (2.3%)

**Table 3:** Changes in Hamilton Depression Rating Scale based on 17 items and Unified Parkinson’s Disease Rating Scale scores at 1 and 3 months vs. baseline.

		Baseline Mean (SD)	1 Month Mean Difference from Baseline (SD)	*p*-Value	3 Months Mean Difference from Baseline (SD)	*p*-Value
HAMD-17	Complete cohort	19.49 (4.03) *n* = 82	−6 (5.10) *n* = 78	*p* < 0.0001	−7.27 (5.48) *n* = 82	*p* < 0.0001
	Safinamide 50 mg	18.50 (2.69) *n* = 22	−3.32 (4.54) *n* = 22	*p* = 0.0003	−4.73 (4.49) *n* = 22	*p* < 0.0001
	Safinamide 100 mg	19.85 (4.39) *n* = 60	−7.03 (5.19) *n* = 56	*p* < 0.0001	−8.02 (5.73) *n* = 60	*p* < 0.0001
UPDRS I	Complete cohort	4.56 (1.82) *n* = 82	−1.32 (1.99) *n* = 78	*p* < 0.0001	−1.5 (2.03) *n* = 82	*p* < 0.0001
	Safinamide 50 mg	4.59 (1.47) *n* = 22	−0.64 (1.91) *n* = 22	*p* = 0.0157	−0.91 (1.76) *n* = 22	*p* = 0.0045
	Safinamide 100 mg	4.55 (1.94) *n* = 60	−1.59 (1.97) *n* = 56	*p* < 0.0001	−1.72 (2.08) *n* = 60	*p* < 0.0001
UPDRS II	Complete cohort	13.59 (6.67) *n* = 82	−2.51 (6.30) *n* = 78	*p* < 0.0001	−2.47 (6.11) *n* = 82	*p* < 0.0001
	Safinamide 50 mg	11.50 (5.20) *n* = 22	−0.36 (5.44) *n* = 22	*p* = 0.4064	−0.23 (5.23) *n* = 22	*p* = 0.4966
	Safinamide 100 mg	14.35 (7.02) *n* = 60	−3.30 (6.65) *n* = 56	*p* < 0.0001	−3.28 (6.45) *n* = 60	*p* < 0.0001
UPDRS III	Complete cohort	22.91 (8.68) *n* = 82	−3.58 (8.56) *n* = 78	*p* < 0.0001	−4.03 (8.95) *n* = 82	*p* < 0.0001
	Safinamide 50 mg	22.00 (8.12) *n* = 22	−0.41 (8.88) *n* = 22	*p* = 0.7722	+0.50 (9.42) *n* = 22	*p* = 0.6723
	Safinamide 100 mg	23.25 (8.92) *n* = 60	−4.8 (8.34) *n*= 56	*p* < 0.0001	−5.70 (8.47) *n* = 60	*p* < 0.0001
UPDRS IV	Complete cohort	3.51 (2.83) *n* = 82	−0.61 (2.61) *n* = 78	*p* = 0.0003	−0.8 (2.53) *n* = 82	*p* < 0.0001
	Safinamide 50 mg	4.64 (2.59) *n* = 22	−0.32 (2.38) *n* = 22	*p* = 0.1839	−0.28 (2.50) *n* = 22	*p* = 0.2482
	Safinamide 100 mg	3.10 (2.82) *n* = 60	−0.76 (2.50) *n* = 56	*p* = 0.0007	−1.00 (2.28) *n* = 60	*p* < 0.0001

HAMD-17: Hamilton Depression Rating Scale based on 17 items. UPDRS: Unified Parkinson’s Disease Rating Scale. SD: Standard deviation. Comparisons were made using the Student’s t-test for paired data. *p* values < 0.05 were considered statistically significant.

**Table 4:** Symptoms related to serotonin syndrome in safinamide-only group vs. safinamide-plus-antidepressants group at 1 and 3 months.

			1 Month			3 Months		
			Safinamide + Antidepressants Group *n* = 42	Safinamide-only Group *n* = 36	*p*-Value	Safinamide + Antidepressants Group *n* = 44	Safinamide-only Group *n* = 38	*p*-Value
Major symptoms	Confusion	*n* (%)	2 (2.6%)	0 (0%)	*p* = 0.564	1 (1.2%)	0 (0%)	*p* = 1.251
	Emotional lability		2 (2.6%)	1 (1.3%)	*p* = 1.021	3 (3.7%)	1 (1.2%)	*p* = 0.627
	Fever		0 (0%)	0 (0%)	-	0 (0%)	0 (0%)	-
	Sweating		3 (3.8%)	1 (1.3%)	*p* = 0.627	3 (3.7%)	0 (0%)	*p* = 0.266
	Myoclonus		0 (0%)	0 (0%)	-	2 (2.4%)	0 (0%)	*p* = 0.565
Minor symptoms	Agitation	*n* (%)	2 (2.6%)	1 (1.3%)	*p* = 1.021	1 (1.2%)	0 (0%)	*p* = 1.251
	Sleep disorders		13 (16.7%)	4 (5.1%)	*p* = 0.053	13 (15.9%)	4 (4.9%)	*p* = 0.054
	Nervousness		15 (19.2%)	4 (5.1%)	*p* = 0.017	13 (15.9%)	5 (6.1%)	*p* = 0.108
	Tachycardia		2 (2.6%)	1 (1.3%)	*p* = 1.021	3 (3.7%)	0 (0%)	*p* = 0.266
	Hyperventilation		0 (0%)	1 (1.3%)	*p* = 0.897	0 (0%)	1 (1.2%)	*p* = 0.894
	Dyspnea		1 (1.3%)	2 (2.6%)	*p* = 0.642	2 (2.4%)	3 (3.7%)	*p* = 0.666
	Diarrhea		0 (0%)	1 (1.3%)	*p* = 0.897	1 (1.2%)	0 (0%)	*p* = 1.251
	Hypertension		0 (0%)	1 (1.3%)	*p* = 0.897	0 (0%)	1 (1.2%)	*p* = 0.894
	Hypotension		0 (0%)	0 (0%)	-	0 (0%)	0 (0%)	-
	Ataxia		0 (0%)	1 (1.3%)	*p* = 0.897	0 (0%)	1 (1.2%)	*p* = 0.894

Data were compared using the Fisher’s exact test. *p* values < 0.05 were considered statistically significant.

## Data Availability

The datasets generated during and/or analyzed during the current study are available from the corresponding author on reasonable request.

## References

[1] Stocchi F., Torti M. (2016). Adjuvant therapies for Parkinson’s disease: Critical evaluation of safinamide. Drug. Des. Dev. Ther..

[2] Borgohain R., Szasz J., Stanzione P., Meshram C., Bhatt M., Chirilineau D., Stocchi F., Lucini V., Iuliani R., Forrest E. (2014). Study 016 Investigators. Randomized trial of safinamide add-on to levodopa in Parkinson’s disease with motor fluctuations. Mov. Disord..

[3] Borgohain R., Szasz J., Stanzione P., Meshram C., Bhatt M.H., Chirilineau D., Stocchi F., Lucini V., Giuliani R., Forrest E. (2014). Study 018 Investigators. Two-year, randomized, controlled study of safinamide as add-on to levodopa in mid to late Parkinson’s disease. Mov. Disord..

[4] Schapira A.H., Fox S.H., Hauser R.A., Jankovic J., Jost W.H., Kenney C., Kulisevsky J., Pahwa R., Poewe W., Anand R. (2017). Assessment of Safety and Efficacy of Safinamide as a Levodopa Adjunct in Patients With Parkinson Disease and Motor Fluctuations: A Randomized Clinical Trial. JAMA Neurol..

[5] Hattori N., Tsuboi Y., Yamamoto A., Sasagawa Y., Nomoto M. (2020). Efficacy and safety of safinamide as an add-on therapy to L-DOPA for patients with Parkinson’s disease: A randomized, double-blind, placebo-controlled, phase II/III study. Parkinsonism Relat. Disord..

[6] Tsuboi Y., Hattori N., Yamamoto A., Sasagawa Y., Nomoto M. (2020). Long-term safety and efficacy of safinamide as add-on therapy in levodopa-treated Japanese patients with Parkinson’s disease with wearing-off: Results of an open-label study. J. Neurol. Sci..

[7] Mancini F., Di Fonzo A., Lazzeri G., Borellini L., Silani V., Lacerenza M., Comi C. (2020). Real life evaluation of safinamide effectiveness in Parkinson’s disease. Neurol. Sci..

[8] Aarsland D., Påhlhagen S., Ballard C.G., Ehrt U., Svenningsso P. (2020). Depression in Parkinson disease-epidemiology, mechanisms, and management. Nat. Rev. Neurol..

[9] Carod-Artal F.J., Ziomkowski S., Mourão Mesquita H., Martínez-Martin P. (2008). Anxiety and depression: Main determinants of health-related quality of life in Brazilian patients with Parkinson’s disease. Parkinsonism Relat. Disord..

[10] Machado-Vieira R., Manji H.K., Zarate C.A. (2009). The role of the tripartite glutamatergic synapse in the pathophysiology and therapeutics of mood disorders. Neuroscientist.

[11] Allain H., Pollak P., Neukirch H.C. (1993). Symptomatic effect of selegiline in de novo parkinsonian patients. The French Selegiline Multicenter Trial. Mov. Disord..

[12] Barone P., Poewe W., Albrecht S., Debieuvre C., Massey D., Rascol O., Tolosa E., Weintraub D. (2010). Pramipexole for the treatment of depressive symptoms in patients with Parkinson’s disease: A randomised, double-blind, placebo-controlled trial. Lancet Neurol..

[13] Rektorova I., Balaz M., Svatova J., Zarubova K., Honig I., Dostal V., Sedlackova S., Nestrasil I., Mastik J., Bares M. (2008). Effects of ropinirole on nonmotor symptoms of Parkinson disease: A prospective multicenter study. Clin. Neuropharmacol..

[14] Korchounov A., Winter Y., Rössy W. (2012). Combined beneficial effect of rasagiline on motor function and depression in de novo PD. Clin. Neuropharmacol..

[15] Zarate C., Machado-Vieira R., Henter I., Ibrahim L., Diazgranados N., Salvatore G. (2010). Glutamatergic modulators: The future of treating mood disorders?. Harv. Rev. Psychiatry.

[16] Richard I.H., Kurlan R., Tanner C., Factor S., Hubble J., Suchowersky O., Waters C., Parkinson Study Group (1997). Serotonin syndrome and the combined use of deprenyl and an antidepressant in Parkinson’s disease. Neurology.

[17] Panisset M., Chen J.J., Rhyee S.H., Conner J., Mathena J., The STACCATO study investigators (2014). Serotonin Toxicity Association with Concomitant Antidepressants and Rasagiline Treatment: Retrospective study (STACCATO). Pharmacotherapy.

[18] Panisset M., Schwied S., Ondo W., Fitzer-Attas C., Chen J.J. (2007). Safety of concomitant therapy with rasagiline and antidepressants in Parkinson’s diesease. Mov. Disord..

[19] Smith K.M., Eyal E., Weintraub D., for the ADAGIO Investigators (2015). Combined rasagiline and antidepressant use in Parkinson disease in the ADAGIO study. Effects on nonmotor symptoms and tolerability. JAMA Neurol..

[20] Aboukarr A., Giudice M. (2018). Interaction between Monoamine Oxidase B Inhibitors and Selective Serotonin Reuptake Inhibitors. Can. J. Hosp. Pharm..

[21] Postuma R.B., Berg D., Stern M., Poewe W., Olanow C.W., Oertel W., Obeso J., Marek K., Litvan I., Lang A.E. (2015). MDS Clinical Diagnostic Criteria for Parkinson’s Disease. Mov. Disord..

[22] Torbey E., Pachana N.A., Dissanayaka N.N.W. (2015). Depression rating scales in Parkinson’s disease: A critical review updating recent literature. J. Affect. Disord..

[23] Tomlinson C.L., Stowe R., Patel S., Rick C., Gray R., Clarke C.R. (2010). Systematic review of levodopa dose equivalency reporting in Parkinson’s disease. Mov. Disord..

[24] Schade S., Mollenhauer B., Trenkwalder C. (2020). Levodopa Equivalent Dose Conversion Factors: An Updated Proposal Including Opicapone and Safinamide. Mov. Disord. Clin. Pract..

[25] Stocchi F., Borgohain R., Onofrj M., Schapira A.H.V., Bhatt M., Lucini V., Giuliani R., Anand R. (2012). A randomized, double-blind, placebo-controlled trial of safinamide as add-on therapy in early Parkinson’s disease patients. Mov. Disord..

[26] Cattaneo C., Müller T., Bonizzoni E., Lazzeri G., Kottakis I., Keywood C. (2017). Long-Term Effects of Safinamide on Mood Fluctuations in Parkinson’s Disease. J. Parkinsons. Dis..

[27] Bianchi M.L.E., Riboldazzi G., Mauri M., Versino M. (2019). Efficacy of safinamide on non-motor symptoms in a cohort of patients affected by idiopathic Parkinson’s disease. Neurol. Sci..

[28] Alborghetti M., Nicoletti F. (2019). Different Generations of Type-B Monoamine Oxidase Inhibitors in Parkinson’s Disease: From Bench to Bedside. Curr. Neuropharmacol..

[29] Cattaneo C., Jost W.H., Bonizzoni E. (2020). Long-Term Efficacy of Safinamide on Symptoms Severity and Quality of Life in Fluctuating Parkinson’s Disease Patients. J. Parkinsons Dis..

[30] Abbruzzese G., Kulisevsky J., Bergmans B., Gomez-Esteban J.C., Kägi G., Raw J., Stefani A., Warnecke T., Jost W.H., SYNAPSES Study Investigators Group (2020). A European Observational Study to Evaluate the Safety and the Effectiveness of Safinamide in Routine Clinical Practice: The SYNAPSES Trial. J. Parkinsons Dis..

[31] Ahmed M.A.A. (2019). A systematic review and meta-analysis of safety and efficacy of safinamide for motor fluctuations in patients with Parkinson’s disease. F1000Resarch.

[32] Gloria Martí-Andrés G., Jiménez-Bolaños R., Arbelo-González J.M., Pagonabarraga J., Carmen Duran-Herrera C., Valenti-Azcarate R., Luquin M.R. (2019). Safinamide in Clinical Practice: A Spanish Multicenter Cohort Study. Brain. Sci..

[33] Baldo B.A., Rose M.A. (2020). The anesthetists, opioid analgesic drugs, and serotonin toxicity: A mechanistic and clinical review. Br. J. Anaesth.

